# Prevention of Diabetic Foot Ulcer: A Neglected Opportunity

**Published:** 2020-05-31

**Authors:** C Miranda, R Da Ros

**Affiliations:** 1 Clinic of Endocrinology and Metabolism Pordenone Hospital-Italy; 2 Clinic of Diabetology Monfalcone Hospital-Italy

We have read with great pleasure the article by Setacci et al.^1^ entitled “Focusing on Diabetic Ulcers” in the last issue of Transl Med UniSa, but we would like to emphasize the importance of prevention.

The diabetes foot ulcer is the most important cause of non traumatic limb amputation. Based on recent data it has been estimated that up to 34% of type 2 diabetes patients may develop diabetic foot ulcer once in their lifetime2. Risk for ulcer recurrence is high, with recurrence rates of 40% in the first year and 65% in the first 3 years, after healing.^2^ The burden of diabetic foot disease is ranked in the top-10 of all medical conditions.^3^

The direct and indirect costs for such a debilitating disease are very high.

Risk factors for the development of foot ulcers are: distal sensory-motor peripheral neuropathy, peripheral arterial disease, previous ulcers and / or amputations.^4^. The most affected are male subjects, with longer duration of illness and low socio-economic level4. Peripheral vascular disease has a heavy impact in the evolution of lesions and is present in approximately 50% of patients with diabetic foot ulcers^5^.

The foot ulcer is a complication of diabetes that can be prevented. In the International working Group on the Diabetic Foot Guidelines 2019^6^ there are five key elements that underpin prevention of foot problems:1) identifying the at-risk foot; 2) regularly inspecting and examining the at-risk foot; 3) Educating the patient, family and healthcare providers; 4) Ensuring routine wearing of appropriate footwear; 5) Treating risk factors for ulceration.

Understanding the factors that place patients with diabetes mellitus at high risk of ulceration, together with the early treatment of risk factors and continuous education on the patient and caregivers^7^ are essential elements to the prevention and management of diabetic foot complications.

Diabetic foot ulceration poses a heavy burden on the patient and the healthcare system, but ulcer prevention remains a neglected opportunity. The best setup for prevention of diabetic ulcer may yet have to be investigated. In 2015, authors of guidelines from the International Working Group on the Diabetic Foot (IWGDF)^8^ underlined that a shift in priority in care and research in diabetic foot disease was needed while other experts argued more evidence from properly-designed studies on this topic^9^.

But after 4 years, the diabetic foot ulcer prevention it’s still neglected in research. Indeed, between 2015 and 2019, 83 RTCs on diabetic foot were published but only two RCTs were conducted on prevention while 72 were on ulcer healing^10^. We agree with authors of guidelines from the International Working Group on the Diabetic Foot (IWGDF).10, researchers and funders have to shift their priorities and we need invest more resources in diabetic foot ulcer prevention in research and clinical practice.
